# Screening and confirmation tests for SARS-CoV-2: benefits and drawbacks

**DOI:** 10.1186/s43088-023-00342-3

**Published:** 2023-01-11

**Authors:** Muhammad Hakimin Shafie, Marie Antony Dass, Hazlam Shamin Ahmad Shaberi, Zainuddin Zafarina

**Affiliations:** 1 Analytical Biochemistry Research Centre (ABrC), Bangunan Inkubator Inovasi Universiti (I2U), Kampus Sains@usm, Universiti Sains Malaysia, Lebuh Bukit Jambul, 11900 Bayan Lepas, Penang Malaysia; 2 School of Life and Environmental Sciences, Deakin University, Waurn Ponds, Geelong, 3216 Australia; 3 Department of Life Sciences, Imperial College London, Exhibition Rd, London, SW7 2AZ UK

**Keywords:** COVID-19, ELISA, LFIA, RT-PCR, SARS-CoV-2

## Abstract

**Background:**

Coronavirus disease 2019 is a pandemic caused by severe acute respiratory syndrome coronavirus 2 (SARS-CoV-2) infection that emerged in late 2019 and has activated an ongoing international public health emergency. SARS-CoV-2 was discovered in Wuhan, China, in December 2019 and rapidly spread to other cities and countries. Currently, SARS-CoV-2 diagnostic tests have relied heavily on detecting viral genes, antigens, and human antibodies. Hence, this review discusses and analyses the existing screening and confirmation tests for SARS-CoV-2, including the real-time reverse transcriptase polymerase chain reaction (RT-PCR), lateral flow immunoassay (LFIA), and enzyme-linked immunosorbent assay (ELISA).

**Main body:**

The illustrations of each testing were presented to provide the readers with an understanding of the scientific principles behind the testing methods. The comparison was made by highlighting the advantages and disadvantages of each testing. ELISA is ideal for performing the maximum population screening to determine immunological capacity, although its inability to provide reliable results on the status of the infection. Recently, LFIA has been approved as a quicker way of determining whether a patient is infected at the analysis time without using particular instruments and non-laboratory settings. RT-PCR is the gold-standard approach in terms of sensitivity and specificity.

**Conclusion:**

However, the combination of LFIA or ELISA with RT-PCR is also proposed in this review to obtain an adequate level of sensitivity and specificity.

**Graphic Abstract:**

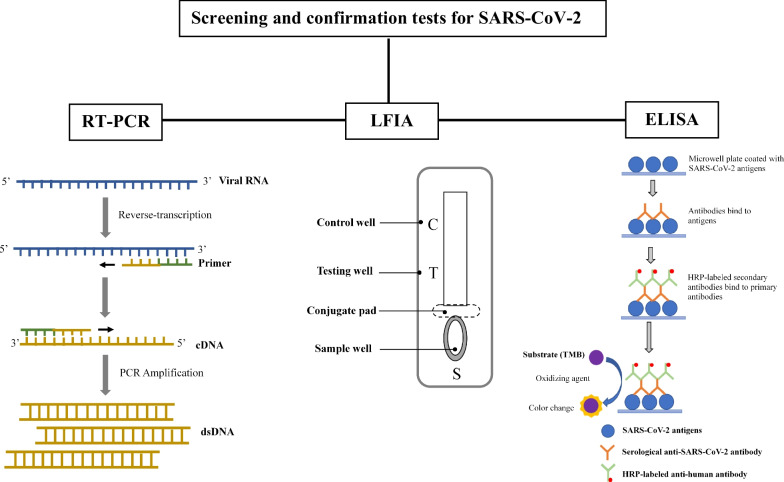

## Background

Coronavirus (CoV) is an enveloped virus with a positive single-stranded RNA genome and is pathogenically ranging from 60 to 140 nm in diameter with spike-like projections on its surfaces giving it a crown-like appearance under the electron microscope [1, 2]. The emergence of the new coronavirus known as SARS-CoV-2 in late 2019 has caused a catastrophic pandemic. The World Health Organization (WHO) named the disease caused by SARS-CoV-2 as “Coronavirus disease 2019 (COVID-19)” on 11 February 2020 and proclaimed it as a global pandemic on 11 March 2020 [3]. This new coronavirus was named as such due to its close genome similarity to the previously reported coronavirus, Middle East respiratory syndrome coronavirus (MERS-CoV) and SARS-CoV, which caused the severe acute respiratory syndrome (SARS) outbreak in 2003 and 2012, respectively [4]. Until now, SARS-CoV-2 has infected almost 237 million people worldwide, with a reported death of around 4.8 million. SARS-CoV-2 has a genome size of approximately 30,000 nucleotides and encodes 29 proteins [5]. Four of these proteins are structural: envelope protein (encoded by the E gene), membrane protein (encoded by the M gene), nucleocapsid protein (encoded by the N gene), and spike protein (encoded by the S gene). The spike protein on the virus surface is responsible for attachment and entry into host cells. This protein binds to the ACE2 receptor on human cells, allowing SARS-CoV-2 to replicate in the respiratory tract, where ACE2 is also expressed and the virus is easily transmitted [6–9]. Diagnostic tests for SARS-CoV-2 often target specific sequences of the viral RNA genome using molecular assays like PCR, or viral proteins like the envelope or spike proteins using immunoassays like lateral flow.

Since SARS-CoV-2 replicates in the upper respiratory tract, respiratory transmission is the most dominant mode of transmission [10]. The virus can be released and spread from an infected individual by coughing, sneezing, and even talking. The respiratory secretions containing the virus can infect other individuals within 1 m. To curb the transmission of COVID-19, WHO has established a few guidelines such as frequent handwashing, wearing a properly fitted mask, and maintaining at least one metre distance from others [11]. Other public health and preventive measures focus on infected individuals, which include community testing, contract tracing, isolation, and quarantines. These focussed measures were effective in decreasing the number of infected cases and mortality rates in many countries [12–17]. Evidence has shown that SARS-CoV-2 is the highest at common locations such as households and other residential sites where sustained and prolonged contacts are made [18]. Thus, early detection is one of the most critical interventions in the fight against SARS-CoV-2 transmission [19].

The symptoms of COVID-19 symptoms range from mild to severe. Mild symptoms include fever, headache, nausea, sore throat, fatigue, loss of smell, and a runny nose. In addition to acute respiratory distress syndrome and respiratory failure, COVID-19 causes acute cardiac injury, systemic inflammation leading to sepsis, multiorgan dysfunction in high-risk patients, and heart failure [20]. Many analyses have shown higher mortality rates in older people and those with comorbidities [21]. Discerning the medical harms of COVID-19, pharmacological approaches are expanding to treat and reduce infection. These include the development of new drugs, the evaluation of the clinical efficacy of older drugs for COVID-19, and the development of vaccines [22–25].

Due to the rapid transmission and severity of COVID-19, sensitive, fast, and robust diagnostic tests for the detection of SARS-CoV-2 are vital for timely clinical intervention and limiting such infection and transmission. Failure to quickly detect infection and curb transmission would affect healthcare systems, particularly burnout of healthcare workers and overflow of the intensive care unit [26, 27]. The current methods used for SARS-CoV-2 detection are mostly based on molecular and serological techniques. Nucleic acid detection is carried out using high-throughput sequencing, RT-loop-mediated isothermal amplification (RT-LAMP), quantitative real-time PCR (qPCR), and reverse transcription polymerase chain reaction (RT-PCR) [28–30]. The diagnosis by real-time reverse transcription PCR (real-time RT-PCR) using upper respiratory tract samples is currently the gold standard for the detection of COVID-19 due to its high sensitivity and specificity. On the other hand, serological methods are cheaper and less tedious. It identifies the types and concentration levels of several immunoglobulins (IgA, IgM, and IgG) in the serum sample [31].

In this study, we intend to discuss the methods being established for screening and diagnosis of COVID-19 that can be useful for early detection of SARS-CoV-2 infection and limit its transmission. The articles were first accumulated from ScienceDirect (https://www.sciencedirect.com/), Scopus (https://www.scopus.com/), and Google Scholar (https://scholar.google.com/) in the most recent year (from 2019 to 2021) using PCR, RT-PCR, NAAT, LFIA, ELISA, reverse transcription polymerase chain reaction, lateral flow immunoassay, enzyme-linked immunosorbent assay, immunoassays for COVID-19, and COVID-19 diagnostic methods as keywords. Next, we synthesise the literature and provide a critique of selected SARS-CoV-2 detection tests, as well as evaluate the pros and cons of each of them. Lastly, the conclusion was made based on the current literature.

## SARS-CoV-2 detection methods

### RT-PCR detection method

#### Basic principles

RT-PCR refers to the real-time reverse transcriptase polymerase chain reaction. RT-PCR for detection and diagnosis has been widely used during the COVID-19 pandemic, including for in-country and international border control, admission and discharge from hospitals or quarantine centres, and epidemiological surveillance in the community [32]. Most importantly, the gold standard for diagnosing this highly infectious disease is RT-PCR [33]. Individuals infected with the virus can be identified for rapid isolation, even before showing any symptoms.

**Fig. 1:**
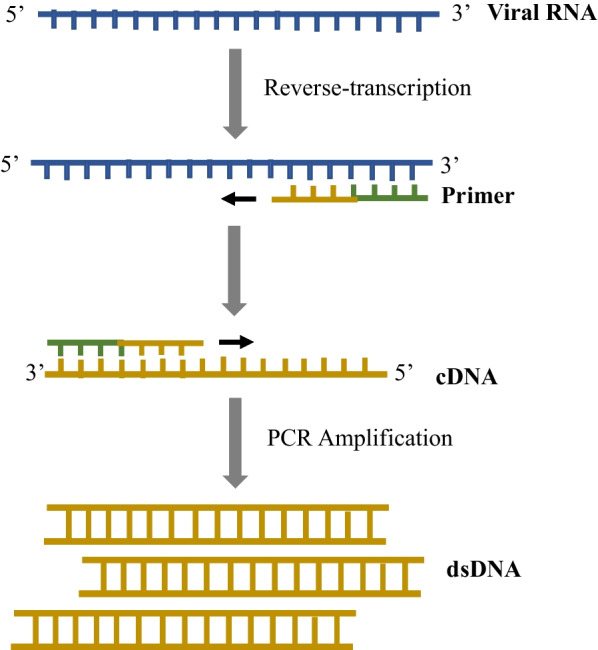
Schematic illustration of RT-PCR. Before RT-PCR is performed, the sample obtained from a tested individual is subsequently subjected to a lysis reaction and RNA purification to extract the viral RNA. If the targeted viral RNA is present, the specific primer binds to a complementary region of the RNA, and reverse transcriptase generates the first-strand complementary DNA (cDNA) using the viral RNA template. Then, PCR cycle at different temperatures to separate the DNA strands (including the first-strand cDNA) allows binding of DNA primers to the template DNA and then allows DNA polymerase enzyme to extend the new DNA strand. Ultimately, this creates more copies of the double-stranded DNA (dsDNA) that allow detection of the viral nucleotide. Adapted from Xu et al*.* [32]

Generally, RT-PCR works by detecting the viral nucleotide by reversely transcribing the viral ribonucleic acid (RNA) into complementary deoxyribonucleic acid (cDNA) and amplifying the cDNA in the presence of reverse transcriptase, deoxyribonucleic acid (DNA) polymerase, specific primers, and free nucleotides (Fig. 1). This requires sophisticated instruments, expensive reagents, and laboratory expertise.

RT-PCR can be qualitative, semiquantitative, and quantitative, where qualitative RT-PCR is the most widely used for the diagnosis of COVID-19 [34]. While a qualitative RT-PCR can detect the absence or presence of the viral nucleotide sequence, a quantitative RT-PCR can also determine the copy number of the viral RNA (known as viral load quantification). This is done by the inclusion of a fluorescent reporter molecule that produces fluorescence following the amplification of cDNA. A sample from an infectious individual with a greater viral load therefore produces higher fluorescence. On the other hand, a semiquantitative RT-PCR can estimate the relative copy number of the viral RNA based on a known ‘housekeeping’ gene. Briefly, the copy number can be estimated from the RT-PCR cycle threshold (Ct) values using standard curves of the Ct values from serial dilution samples and the estimated viral loads.

#### Applications, advantages, and disadvantages

The timeline of positivity by RT-PCR varies across the disease stage, where an infected individual is most likely to be detected positive during early symptom onsets [35]. The interpretation of a positive result needs to be understood as this relates to implications such as determining the clinical cure status of an admitted patient and determining the infectiousness of a tested individual for isolation purposes. In general, the purposes of testing may be (a) a test of infection to detect an infected individual, (b) a test of infectivity to measure if an individual is contagious or not, or (c) a test of cure to observe if an infection has subsided.

In terms of tests for infection, RT-PCR is useful for confirming cases even before symptom onset due to its better detection rate (i.e. the ability to yield a positive result) than other diagnostic tools. However, infected individuals who are asymptomatic or presymptomatic may be unlikely to opt for RT-PCR testing due to its invasive swabbing method, costly, and not point-of-care nature. Given the relatively long mean incubation period of 5–6 days and the major infections that occur during the presymptomatic phase for the currently dominant Omicron variant, RT-PCR may not be the ideal test for rapid screening purposes [36]. Nonetheless, RT-PCR remains the best to detect infection, such as for confirming individuals with symptoms or close contacts, community surveillance, or testing for international travel. Even so, the pitfalls of RT-PCR for detecting infection include its inability to distinguish between COVID-19 reinfection or reactivation (described as recurrence) from prolonged viral shedding from a previous initial infection that may still yield a positive RT-PCR result long after recovery [34]. By assessing the recurrence in patients with a late repeat of positive RT-PCR based on clinical assessment, epidemiological analysis, or viral genome sequencing, at least two studies have confirmed that COVID-19 recurrence does occur despite being very rare [37, 38]. In both studies, most of the late repeat positive SARS-CoV-2 RT-PCR detected was inconsistent with the true recurrence. Thus, RT-PCR positivity should be taken with context before deducing an infection in the tested individuals, especially for those with a known history of COVID-19 infection.

Next, to decide whether an infected individual needs to be isolated or discharged from quarantine, tests of infectivity can be carried out through quantitative or semiquantitative RT-PCR. Infectivity is measured by looking at the Ct values from RT-PCR which generally correlate inversely with viral load. Indeed, studies investigating the association between Ct values and infectivity reported higher cell culture positivity (defined as an infectivity marker) for lower Ct values [39]. Thus, determining a cut-off Ct value is essential to determine which range of Ct values is safe to categorise an individual as infectious or otherwise. As mentioned by Rao et al*.* [39] and Han et al. [40], different quantitative RT-PCR kits can use different standard curves to quantify Ct values. The same Ct values of different kits can indicate a different amount of viral load. Therefore, the cut-off Ct values for different kits are different and should not be standardised for a reliable diagnosis.

Lastly, tests of cure may be conducted to observe the patient’s status for isolation discharge purposes. However, some patients can still be detected as positive by RT-PCR of nasopharyngeal swab more than 8 weeks after symptom onset due to prolonged viral shedding, even though the median time for clinical recovery is only about 14–15 days after the symptom onset [34]. Therefore, current guidance has ruled out the need for clearing RT-PCR tests to discharge COVID-19 patients [41].

#### Questioning the gold-standard status of RT-PCR for COVID-19 diagnosis

The status of RT-PCR as a gold standard for COVID-19 diagnosis was previously challenged by a study by Wikramaratna et al. [42] reporting that the probability of an individual with infection being detected positive decreases the later they are tested after their symptom onset. Another study evaluated the sensitivity of nasopharyngeal swab RT-PCR using high clinical suspicion of COVID-19 as the reference standard [43]. They reported that the false negative rate of SARS-CoV-2 RT-PCR is moderate (47.3%, 95% confidence interval (CI) 44.4–50.3%) in symptomatic patients. However, severe symptoms are linked to a larger viral load and therefore are supposedly more likely to be detected [44]. In particular, the late delay of the test from symptom onset could not be the cause of the moderate false negative rate of RT-PCR reported since there was no significant difference between the delay time and the false negative rate in this study. In addition to the late delay of the test, other studies found several reasons for the reduced sensitivity of RT-PCR. First, different kits can significantly differ in their lowest detection concentration, as shown in a study conducted by Zhou et al*.* [45] on various RT-PCR SARS-CoV-2 detection kits, including the Centers for Disease Control and Prevention (CDC) and WHO. Second, the sensitivity may also be reduced because of mismatches in target genes at the primer or probe binding sites. Mismatches could be due to mutations emerging in new variants or primer/probe problems, such as primers that contain degenerate nucleotides [45, 46].

Although RT-PCR is widely used to categorise infectious and non-infectious individuals by relying on the general correlation between Ct values and infectivity, there are several issues with it. Firstly, an individual's viral load can be different among different clinical specimen types such as saliva and throat swab. Therefore, Ct values for infectivity should be used for specimen types that consistently report the highest viral load. A study by Sharma et al*.* [47] reported that the combined nasopharyngeal and oropharyngeal swab had the lowest average Ct value compared to the nasopharyngeal swab, the oropharyngeal swab, and the sputum. Thus, perhaps a combined nasopharyngeal and oropharyngeal swab would be a good specimen standard for associating Ct values with infectivity. Also, the use of RT-PCR Ct value as the proxy of infectivity is uncertain regarding vaccination status. To assess whether a similar viral load between vaccinated and unvaccinated individuals has similar infectivity, Riemersma et al*.* [48] cultured samples with low Ct values (< 25) from vaccinated (*n* = 39) and unvaccinated (*n* = 17). They did not observe differences between the two groups as infectious SARS-CoV-2 was detected in nearly all cultured samples. In contrast, a small study by Ke et al*.* [49] and a preprint of a larger study by Shamier et al*.* [50] reported that for a given viral load, samples from vaccinated individuals observed a lower probability of culture positivity than samples from unvaccinated individuals. Therefore, to better use the Ct value as a proxy of infectivity, standardisation of the specimen type and a greater understanding of the association of viral load and infectivity for vaccinated individuals are needed.

Accounting for the mentioned problems associated with RT-PCR, we suggest that a negative result from RT-PCR needs to be interpreted with context and not be blindly taken as a true negative. The reported reduced sensitivity and moderate false negative rate need to be accounted for when using RT-PCR for diagnosing COVID-19 or as a reference standard in research [51]. Clinically, it is relatively safer not to rule out COVID-19 in suspicious patients with symptoms from only one negative RT-PCR result [43]. Practically, the sensitivity of an RT-PCR kit should be continuously assessed especially for currently prevalent variants, and only ultra-high sensitive kits should be used for mixed sample testing (i.e. samples from different individuals mixed in a tube for testing) [45].

Alternatively, other appropriate reference standards could be clinical suspicion, computed tomography (CT) imaging, serology, or Droplet Digital PCR (ddPCR), which showed a higher sensitivity than RT-PCR [52–54]. Clinical suspicion involves the examination of clinical experts on patients while CT imaging involves a chest CT scan to check for typical radiological findings that characterise SARS-CoV-2 infection in the lung of patients. Meanwhile, ddPCR amplifies nucleotide fragments differently from RT-PCR by amplifying them in partitioned reaction vessels. Then, ddPCR does not use a standard curve to quantify the concentration of target molecules but counts them directly by enumerating positive droplets. Next, some selected serological tests will be further discussed in the following subsections.

### Lateral flow immunoassay (LFIA) detection method

#### Basic principles

**Fig. 2:**
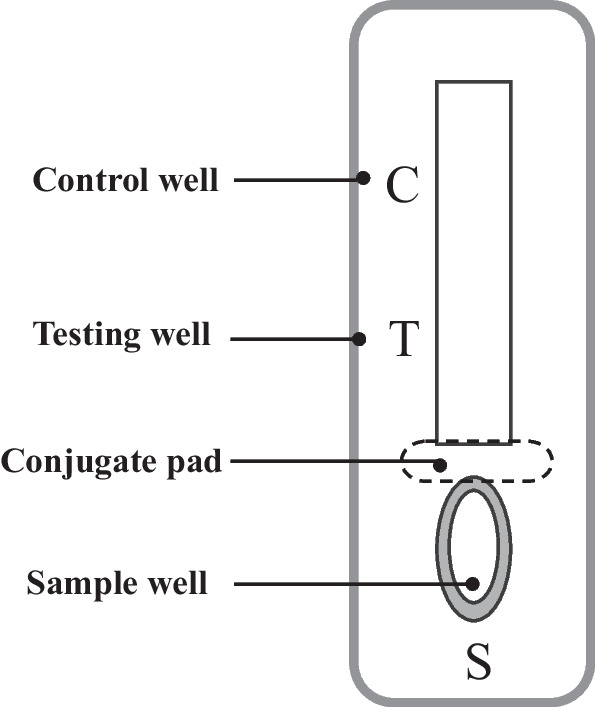
A general configuration of the LFIA test strip. To initiate LFIA, the sample of interest is applied to the sample pad and then flows through the sample pad to the conjugate release pad, rehydrating the analyte-specific antibodies or the attached virus antigen. The associated antibody or antigen, together with its target, flows towards the detector zone of the strip after the contacts. As the complex moves up the membrane, it passes the immobilised analyte-specific antibody, which recognises and binds to it, resulting in a coloured line. The control antibody recognises and captures the control well portion of the antibody detector, resulting in a coloured line that eventually serves as a control. Results are interpreted on the reaction matrix as the presence or absence of lines of captured conjugate, read either by Ching [56]

The lateral flow immunoassay (LFIA) has gained increasing interest in diagnostic applications due to its numerous advantages that meet the WHO guidelines for diagnostic tests [55]. LFIA (Fig. 2) is generally identified as ASSURED: affordable, sensitive, specific, user-friendly, rapid and robust, equipment-free, and deliverable to end-users [56, 57]. It is often used to identify a target material in a liquid sample as a cost-effective alternative to specialised and expensive equipment. These tests are commonly used in food and environmental health safety, clinical laboratories, hospitals, physicians, veterinary medicine, disease identification, agriculture, molecular diagnostics, and theragnostic [56, 58].

#### Applications, advantages, and disadvantages

In recent years, LFIA has gained considerable attention due to its advantages such as ease of use, short analysis time, economical, high sensitivity, specificity, and stability as point-of-care (POC) testing [58]. There are a substantial number of studies that have used LFIA for various novel detections and tests, for example, the detection of pathogenic microorganisms, detection of human hormones or related proteins, parasitic disease diagnosis, blood and urine drug monitoring, testing of pesticides or residues of veterinary drugs in food, and for environmental detections [57, 59–65]. Since the SARS-CoV-2 outbreak, RT-PCR and CT imaging have been used as a gold standard for early-stage infection screening and diagnosis. However, RT-PCR test's sensitivity can vary depending on viral load, sample types, sampling techniques, and time of infection [52]. Recently, novel LFIAs have been developed as a screening tool, or as a complementary test for the diagnosis of SARS-CoV-2 as these tests can be used easily as POC tests. The performance of different LFIAs for COVID-19 detection is summarised in Table 1.

**Table 1:** Comparison of different types of LFIA tests developed for COVID-19 detection

Types of LFIA	No. of positive patient samples^a^	No. of negative patient samples^b^	No. of other samples^c^	Sample type	Sensitivity (%)	Specificity (%)	Reference
NG-Test IgG-IgM LFIA	82	57	25	Human serum	100%	(98%) compared to ELISA (95.8%)	[52]
Lanthanide-doped polystyrene nanoparticles (LNP) LFIA	7	12	ND	Human serum	ND	ND	[66]
Surface – enhanced Raman scattering-based LFIA (SERS-LFIA)	19	49	ND	Human serum	100%	100%	[67]
QB-based LFIA	69	53	ND	Human serum	97.1%	100%	[68]
UK-RTC Abc-19 Rapid Test LFIA	304	350	ND	Human serum	97.70%	100%	[69]

*LFIA* lateral flow immunoassay, *ND* not done, *RT-PCR* reverse transcription polymerase chain reaction

^a^SARS-CoV-2 RT-PCR results are positive

^b^Healthy controls

^c^Banked samples from patients with additional viral illnesses, including SARS-CoV-2

Until now, no study had described the diagnostic performance in NG-Test LFIA until Nicol et al. [52] used it for the diagnostic of SARS-CoV-2. Outstanding 100% sensitivity and 98% specificity were observed in the performance of the NG-Test LFIA for IgG, 15 days after the onset of symptoms compared to ELISA (95.8%). Over the past few years, many LFIA has been using conventional fluorescent dye-based LFIA as a form of quantitative and semiquantitative detection [70]. However, a conventional fluorescent dye is not an ideal reporter because they have poor stability and are liable to photobleaching. Therefore, to overcome these problems, Chen et al. [66] created an LFIA that detects anti-SARS-CoV-2 IgG in human serum by using lanthanide-doped polystyrene nanoparticles (LNPSs). Liu et al. [67] proposed a real-time two-channel surface-enhanced Raman scattering (SERS)-based LFIA biosensor to simultaneously detect anti-SARS-CoV-2 IgM/IgG with high sensitivity with the goal of increasing the sensitivity of the LFIA strip that can potently improve the detection rate of SARS-CoV-2. In his investigation, the detection sensitivity of the suggested method for virus-specific IgM and IgG was 800 times higher than the standard Au-based LFIA method, based on SERS signal intensities of corresponding test zones of the LFIA strip. Colloidal gold nanoparticles-based LFIA (AuNP-LFIA) is recognised as a rapid development for SARS-CoV-2 detection using serological tests. However, the use of AuNP-LFIA to diagnose COVID-19 has turned debatable due to its low sensitivity and high false negative rates [68]. Therefore, Zhou et al. [68] designed and developed quantum nanobeads (QBs)-based LFIA to identify SARS-CoV-2 using human serum as the specimen has demonstrated great potential in enhancing targeted detection. Another novel UK-RTC AbC-19 LFIA was tested by Robertson et al. [69] where Roche and Abbott LFIA were used to determine SARS-CoV-2 infection and resolve the controversy of commercial LFIA that lacked in the detection of IgG antibodies up to 90 days. UK-RTC AbC-19 LFIA showed rapid and robust performance as a POC test in SARS-CoV-2 infection detection with a sensitivity of 97.70% (95% CI 95.31–99.07) and specificity of 100% (95% CI 98.95–100) based on 304 positive and 350 negative patient samples.

In general, an antigen-based LFIA test is convenient for whole blood, serum, and plasma, which also decreases the risks of exposure to viral samples when diagnosing patients with COVID-19. Fingerpricking to obtain the blood of an individual makes this test user-friendly, as no professional personnel is required. These tests can be carried out in any public space at any desirable time. Furthermore, the LFIA test allows the screening of a large number of asymptomatic carriers in a short period. At the moment, with the emergence of the dominant highly transmissible Delta and Omicron variants having a test tool that allows large and quick screenings may appear to be the best option. COVID-19 can be distinguished from other respiratory viral infections, such as influenza, with the help of LFIA [67]. In addition to that, LFIA impacts low-income countries due to its fast turnaround and lower prices.

There are a few limitations of LFIA which are essential to consider. Firstly, due to the low sensitivity of the tests, an antibody response to SARS-CoV-2 could result in a false negative result in the early stages of infection. Sensitivity can also decrease when tested against patients with low or mild symptoms of SARS-CoV-2 infection, as they are only capable of generating a small amount of antibodies. Aside from that, there are no IgG standards available, so assays cannot be improved from semiquantitative to accurate quantification [67]. Moreover, if the amount of antigen in a sample is below the limit of detection (LOD), there is a strong possibility of negative results [71]. Despite the anticipated limitations, LFIA plays a significant role in managing public health and is an excellent surveillance tool to monitor those potentially infected with SARS-CoV-2. LFIA indeed has the ability to screen a larger number of asymptomatic individuals in a short time as compared to RT-PCR. Nevertheless, considering the limitations of antigen tests, LFIA cannot replace RT-PCR for confirmatory test tools, but LFIA is a great screening test tool or a complementary test strip. Due to the potential of LIFA elaborated earlier, we urge future studies to focus on improving the sensitivity and specificity to remove this setback of LFIA.

### Enzyme-linked immunosorbent assay (ELISA) detection method

#### Basic principles

**Fig. 3:**
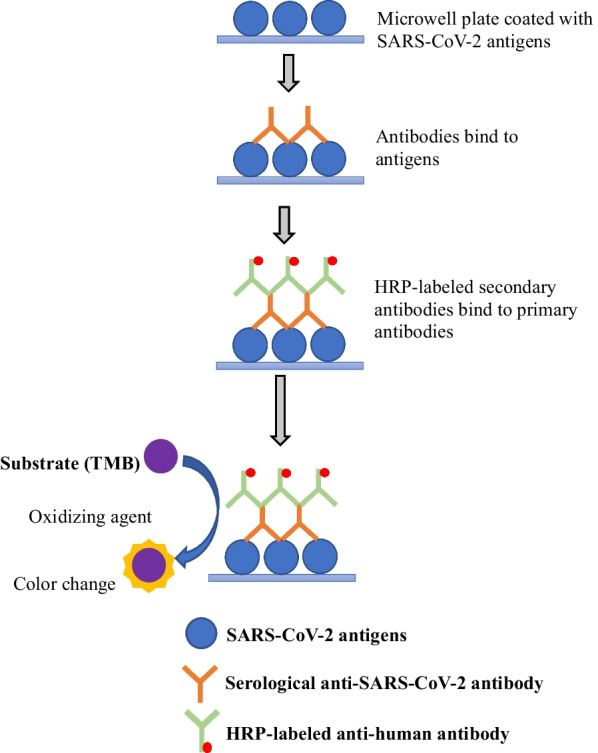
Schematic illustration of ELISA. Antigens from the sample being tested are attached to a surface. The antigens will bind to the matching antibodies applied onto the surface. Antibodies are attached to an enzyme, and any unbound antibodies are eliminated. The enzyme's substrate is added in the final stage. If binding occurs, the subsequent reaction generates a visible indication, most typically by a change in colour change. Adapted from Xu et al*.* [32]

Enzyme-linked immunosorbent assay (ELISA) is a bioanalytical technology that uses an antigen–antibody interaction to detect and quantify an analyte. In 1971, Engvall and Perlmann coined the term ELISA, which provided a method for analysing the concentration of Immunoglobulin G in human serum. Until now, the popularity of ELISA has remained [72]. The solid-phase (e.g. microwell plate) enzyme immunoassay is employed for ELISA to reveal the existence of a ligand (e.g. protein) in a liquid sample using antibodies directed against the protein to be assessed. The procedure begins with a solid phase in which the hypothesised antigen (or antibody) is bound, followed by the addition of serum for testing. If antibodies associated with the immobilised antigen or antibody were present in the serum sample, they would bind to the solid phase [73]. According to Wu et al. [73], the ‘lock and key’ mechanism occurs when the antibody contains a chemical ‘lock’, and the antigen comprises a particular chemical group or ‘key’ that fits this lock (Fig. 3). Antibodies are linked to the antigen in a well in an immunoassay, allowing their concentration to be measured. How the bound analyte is read makes ELISA so superior to other techniques for immunoassays.

#### Applications, advantages, and disadvantages

ELISA is commonly used to detect and quantitate molecules (i.e. antibodies, antigens, hormones, cytokines, and peptides) and study their molecular interactions [74]. The desired analyte concentration is determined in an ELISA by using a generic monoclonal antibody that has been conjugated to an enzyme. With the addition of a simple substrate, the enzyme changes colour, allowing the concentration of bound analytes to be read. ELISA becomes a cheap, easy, and direct technique as a result of the more accessible reading process, with only negligible amounts of specificity and sensitivity being lost. The benefit of this assay is due to its strong specificity, high sensitivity, non-radioactivity, speed, and ease of simultaneous testing even with large quantities of samples. Stadlbauer et al*.* [75] designed a two-stage ELISA protocol to evaluate human antibody responses to the recombinant receptor-binding domain of the spike protein or the full-length spike protein of SARS-CoV-2. A high-throughput screening of samples in a single-serum dilution against the receptor-binding domain was performed in the first stage, followed by a confirmation ELISA against the full-length spike protein in the second stage. Studies that have used ELISA in the antibody detection of SARS-CoV-2 are listed in Table 2.

**Table 2:** List of ELISA applications in the detection of SARS-CoV-2 (adapted and modified from Espejo et al*.* [76])

Antigen	No. of positive patient samples^a^	No. of negative patient samples^b^	No. of other samples^c^	Sensitivity (%)	Specificity (%)	Comments	References
RBD	40	30	207	100	100	Total antibody	[77]
S1	5	120	ND	95	98	IgG	[78]
RBD and SP	53	ND	ND	IgA: 92, IgG: 96, and IgM: 98	99.3	> 10 days after PCR-proven infection	[79]
SP and RBD	16	0	50	ND	ND	Included isotype expression	[80]
RBD	30	10	72	93	100	Total antibody	[81]
S1	30	10	72	67	96	IgG	
S1	30	10	72	93	93	IgA	
NP	208	150	140	ND	ND	IgM, IgG and IgA timeline	[82]
RBD	161	213	ND	93	99	Total antibody timeline	[83]
RBD	143	213	ND	83	99	IgM timeline	
NP	112	197	ND	65	99	IgG timeline	
SP, S1, NP and RBD	41	76	192	ND		IgG and IgA, PRNT	[84]
NP	214	100	ND	80	100	IgM and IgG timeline	[85]
RBD	214	100	ND	82	100	IgM and IgG timeline	
NP	80	100	ND	89	100	IgG	[86]
RBD	80	300	ND	98	100	Total antibody	
RBD	80	300	ND	93	100	IgM	
NP	238	120	ND	82	94	Versus PCR results, timeline	[87]
NP + RBD	12	6	ND	ND	ND	Included quantitative titres	[88]
NP	16	ND	ND	IgG:94 and IgM:88	ND	IgM and IgG	[89]
RBD	16	ND	ND	IgG:100 and IgM:94	ND		
Unknown	63	35	ND	87	100	Compared to LFIA	[90]
RBD	76	ND	150	99	99	Total antibody	[91]
RBD	76	ND	150	89	99	IgM	
S1	43	ND	161	82	99	IgG	
S1	76	ND	161	97	94	IgA	
SP and NP	130	16	ND	ND	ND	IgG and prognosis	[92]
S1	128	10	72	84	88	IgG, IgA and timeline	[93]
RBD	130	108	52	> 80	> 95	IgM/IgG 16 after symptoms	[94]
S1	69	412	ND	97	98	Included asymptomatic study	[95]
S1 and RBD	77	60	40	Presented by timeline	83 IgA and 97–98 others	IgA, IgG, IgM, and total antibody	[96]

*ND* not done, *LFIA* lateral flow immunoassay, *PRNT* plaque reduction neutralisation test, *NP* nucleocapsid protein, *RT-PCR* reverse transcription polymerase chain reaction, *RBD* receptor-binding domain, *SP* spike protein, *S1* subunit of SP contains the RBD needed for binding to the host angiotensin-converting enzyme 2 (ACE2) receptor

^a^SARS-CoV-2 RT-PCR results are positive

^b^Healthy controls

^c^Banked samples from patients with additional viral illnesses, including SARS-CoV-2

Giri et al*.* [97] stated that ELISA assays based on the antibodies have 70–95% sensitivity. Many studies have focussed on ELISA using SP antigen or RBD antigen (Table 2), while few studies focussed on additional necessary peptides that may also be the target of the humoral immune response. In this early research, ELISA using NP and RBD antigens was the most used. Although the timing of sample acquisition altered the sensitivity of the assays, the use of total antibody detection consistently gave a high overall sensitivity than others that were based on a single or few antibodies only [77, 81, 83, 86, 91]. Lassauniere et al*.* [81] reported that the sensitivity of IgA ELISA exhibited higher sensitivity than IgG, with the value of 93% and 67%, respectively. However, research conducted by Peterhoff et al*.* [79] showed that the sensitivity of IgG (96%) was higher than IgA (92%) and IgM was the highest, with a value of 98% for more than 10 days after PCR-proven infection. This is because some antibodies, such as IgG, are correlated with the time of infection. As stated by Peterhoff et al*.* [79], IgG increased early only in hospitalised patients with severe COVID-19 and would reach the threshold an average of 29 days after symptom onset. Essentially, although ELISA may help assess antibody titres and selective isotype detection, it is time-consuming and inappropriate for point-of-care testing.

ELISAs are not as precise as tests identifying viral RNA to detect COVID-19 infection in the early stage. This is because the use of ELISA in the early stage of infection to diagnose the antibody response to the SARS-CoV-2 virus could exhibit false negative results, as the patients' antibodies mostly develop in the recovery phase, which is the 2nd week of infection [98]. As mentioned by Deeks et al*.* [99], ELISA tests have too low a sensitivity to be employed as a primary diagnostic technique for COVID-19 in the 1st week after symptoms onset. However, they may still be useful in individuals whose antibodies are present later, when RT-PCR tests are not performed or there are negative results. Results could also be false positive by preceding COVID-19-like diseases that muddle the specificity of the antibody response. Regardless of its high sensitivity, an antigen used in a diagnostic assay may not be the best protein to target for diagnostics if it is highly conserved throughout a wide range of SARS-CoV-2 in different animals, such as that occurring in SARS-CoV, as this may reduce its specificity [100]. Multiple antibodies should be detected to avoid erroneous results [97], and at an early stage of SARS-CoV-2 infection, another biomarker should be included to improve the sensitivity of ELISA assays [101]. Other than that, using ELISA positivity as a confirmation test for vaccinated individuals is uncertain as the result could be false positive as vaccination also produces a positive antibody test. Research by Tretyn et al*.* [106] reported that non-infected individuals with the first dose of mRNA vaccine (2 weeks later) did not show a significant difference from COVID-recovered individuals for IgG concentration. Hence, other existing COVID-19 testing such as RT-PCR could complement the ELISA test antibody for SAR-CoV-2 detection.

Generally, ELISA is appropriate for situations based on the following: (a) COVID-19 infection is suspected in patients who have a negative RT-PCR result but no significant epidemiological or clinical evidence; (b) patients who were detected after their symptoms onset for longer than 7 days; (c) contact tracing; (d) assessing possible immunity and the probability of protection against reinfection; and (e) seroepidemiological research to a better understanding on COVID-19 circulation in the community [74]. As stated by Peterhoff et al*.* [79], ELISA is required to assess whether a patient had recovered from a previous infection. Therefore, it was suggested that the ELISA could be used to discharge recovered patients who are vulnerable, such as those admitted to the intensive care unit (ICU). The advantages and disadvantages of RT-PCR, LFIA, and ELISA for COVID-19 are summarised in Table 3.

**Table 3:** Advantages and disadvantages of RT-PCR, LFIA, and ELISA

Test	Advantages	Disadvantages
RT-PCR	Ease of use Can measure infectivity Can detect an infection at its early phase Has the highest accuracy compared to LFIA and ELISA in detecting infections	Laborious and requires sophisticated instruments Requires expertise Take few days to get results Swabbing method is Invasive Costly Ct value as a proxy for infectivity has problems such as standardised specimen and uncertainty regarding vaccination status DNA-dependent (problems can arise from different type of specimen used, degenerate nucleotides in primers, and rapid evolution of SARS-CoV-2) Can give positive results even weeks after recovery Cannot distinguish reinfection and new infection
LFIA	Ease of use Short analysis time User-friendly and does not require any expertise Allows the screening of a large number of asymptomatic carriers in a short period Relatively cheaper	Convenient for whole blood, serum, and plasma Cannot be used to detect COVID-19 infection in the early phases Can distinguish infectious and non-infectious individual but not infected and non-infected individual
ELISA	High sensitivity for later stages High specificity due to the reaction between antigen and antibody Useful in individuals whose antibodies present later	Cannot be used to detect COVID-19 infection in the early phases

## Specimens for SARS-CoV-2 detection

Appropriate type and quantity of specimens have a significant impact on the accuracy of diagnostic tests such as RT-PCR, LFIA and ELISA. RT-PCR is generally applicable within a wide range of sample types for the diagnosis of many diseases, including COVID-19. According to several studies, SARS-CoV-2 RNA has been found in a variety of clinical specimens, including upper and lower respiratory tract specimens; faeces; blood serum; blood plasma; anal swab and corneal secretion [47, 103, 104]. However, a comparative analysis by Sharma et al*.* [47] indicates that the optimal clinical specimens for diagnosis need to be accessible, non-invasive, have fewer risks to the healthcare workers who collected the specimens and have good viral loads for a higher detection rate. Although viral detection in specimens, such as blood, urine, and faeces, indicates a possible transmission route of the virus, the detection rate using these specimens is low and inconsistent, since the main established transmission route mode of the virus is by respiratory droplets and aerosols.

Several lower respiratory tract specimens with high detection rates such as bronchoalveolar lavage, fibrobronchoscope brush biopsy, and endotracheal aspirate are not ideal due to the invasive sample collection method and high risk of infecting healthcare workers. Meanwhile, sputum was recommended in several studies and meta-analyses due to its high detection rate in hospitalised symptomatic patients [98, 105]. Sharma et al*.* [47] argued that it is not generally ideal, especially for patients with dry cough and asymptomatic patients who are not able to produce sputum. They also found a low detection rate for sputum specimens in their study to support the claim.

Upper respiratory tract specimens, particularly oropharyngeal and nasopharyngeal swabs, have been widely used for community screening and discharge from hospitals. The gold-standard specimen to identify SARS-CoV-2 is a nasopharyngeal swab by RT-PCR, in part due to its high detection rate consistently found in different studies [41, 97, 98], including a meta-analysis by Czumbel et al*.* [106]. Another meta-analysis by Lee et al*.* [107] also found that the positive detection rates by nasopharyngeal swab alone are higher than nasal or oropharyngeal swab alone. Importantly, combined nasopharyngeal swab with nasal or with oropharyngeal swab were found to have a higher detection rate than nasopharyngeal swab alone [47, 107].

Saliva specimen is the least invasive and easiest to collect compared to other specimens and allows for self-collection. Several meta-analyses have suggested that saliva is a comparable alternative specimens to nasopharyngeal swabs. An early meta-analysis by Czumbel et al*.* [106] suggested saliva by RT-qPCR as a promising candidate for detection as it has a slightly lower sensitivity of 91% (95% CI 80–99%), compared to the nasopharyngeal swab, which has a sensitivity of 98% (95% CI 89–100%) when tested in hospitalised patients with confirmed COVID-19. Addressing the imperfect nasopharyngeal swab nucleic acid amplification testing (NAAT) as a reference standard, a more recent meta-analysis by Butler-Laporte et al*.* [108] also supported similar sensitivity and specificity of saliva specimen to nasopharyngeal swab, especially in ambulatory settings. However, another recent meta-analysis by Lee et al*.* [19] reported that the dual positive of nasopharyngeal swab and saliva is significantly lower than the single positive in either specimen. This finding indicates a poor agreement between the detection of the nasopharyngeal swab and saliva. Therefore, with the inconsistent findings, results obtained from RT-PCR using saliva, especially the negative results, need to be cautiously interpreted.

On the other hand, immunoassays for COVID-19 detection, such as LFIA and ELISA, typically use antibodies in the assay serum that react with SARS-CoV-2 protein in the specimen. According to MacMullan et al*.* [109], the Centre for Disease Control and Prevention has added several upper respiratory tract specimen types as a list of options for recommended sample types. The variety of specimen selections allows for more widespread testing, and the simplest collection methods would allow for widespread and frequent testing. Although serum is the most common sample used to identify antibodies produced against numerous infectious diseases, dried blood spots and saliva samples have also been used successfully [100]. Saliva samples are particularly appealing because they are non-invasive and easy to obtain, allowing self-collection and large-scale testing [103]. As mentioned by Hettegger et al*.* [110], IgG antibody profiles in blood and saliva are similar, with antibody titres for Hepatitis B corresponding well between plasma and saliva.

## Conclusions

Although RT-PCR, antigen-based LFIA, and ELISA have been used during the pandemic, many issues for the respective detection tools need to be addressed. The use of each tool as a screening and confirmatory test needs to be in line with the correct interpretation of the results, within the clinical and epidemiological context. RT-PCR is widely used as a confirmatory test, and many studies have addressed its reliability and sensitivity to detect SARS-CoV-2 infection and to measure the infectivity by Ct values. On the other hand, for immunoassays such as antigen-based LFIA and ELISA, their positivity only increases over time from symptom onset, with a large false negative during the early stages of infection. Therefore, enhancing immunoassays with higher sensitivity detection during the early phase of infection is necessary if they are to be used to detect early infection. With the Omicron variant that is even more transmissible in asymptomatic and presymptomatic individuals, we could not afford to have a screening assay that has a low diagnostic sensitivity. To obtain an adequate level of sensitivity and specificity, combining clinical, molecular, and serological tests may be the best as they can complement each other.

## Data Availability

Not applicable.

## Abbreviations

COVID-19: Coronavirus disease 2019
SARS-CoV-2: Severe acute respiratory syndrome coronavirus 2
RT-PCR: Real-time reverse transcriptase polymerase chain reaction
LFIA: Lateral flow immunoassay
ELISA: Enzyme-linked immunosorbent assay
CoVs: Coronavirus
MERS-CoV: Middle East respiratory syndrome coronavirus
WHO: The World Health Organization
FDA: Food and Drug Administration
EUA: Emergency use authorization
RNA: Viral ribonucleic acid
cDNA: Complementary deoxyribonucleic acid
DNA: Deoxyribonucleic acid
Ct: Cycle threshold
CDC: Centers for Disease Control and Prevention
CT: Computed tomography
ddPCR: Droplet Digital PCR
